# Red Complex Periodontal Pathogens and Their Potential Role in Colorectal Carcinogenesis: A Narrative Review

**DOI:** 10.3390/ijms262010012

**Published:** 2025-10-15

**Authors:** Ursa Potocnik Rebersak, Rok Schara

**Affiliations:** 1 Department of Oral Diseases and Periodontology, University Medical Center Ljubljana, 1000 Ljubljana, Slovenia; ursa.potocnik.rebersak@kclj.si; 2 Faculty of Medicine, University of Ljubljana, 1000 Ljubljana, Slovenia

**Keywords:** periodontal disease (PD), red bacterial complex, colorectal carcinoma (CRC), *Porphyromonas gingivalis*, *Tannerella forsythia*, *Treponema denticola*

## Abstract

Periodontal disease (PD), a chronic inflammatory condition driven by oral microbial dysbiosis, is increasingly implicated in systemic diseases, including colorectal cancer (CRC). The “red complex” bacteria—*Porphyromonas gingivalis*, *Tannerella forsythia*, and *Treponema denticola*—play a central role in PD progression and exhibit virulence factors that promote inflammation, immune evasion, and epithelial colonization. A literature search in PubMed, Google Scholar, and ScienceDirect (English and Slovenian, up to September 2025) identified 12 eligible studies. Only original clinical, in vivo, or in vitro research directly addressing red complex pathogens and colorectal cancer was included. The search results showed that most of the literature focuses on the association between *Porphyromonas gingivalis* and CRC, particularly its role in tumor immune evasion, alteration of the gut microbiota, creation of a pro-inflammatory microenvironment, and promotion of carcinoma cell proliferation. Infection with *Porphyromonas gingivalis* has also been linked to poorer cancer prognosis. The other red complex bacteria are primarily mentioned in the context of generating a pro-inflammatory microenvironment and contributing to chronic inflammation, which supports tumor growth and survival.

## 1. Introduction

Periodontal disease (PD) is a chronic, multifactorial inflammatory condition triggered by dysbiosis within the dental plaque biofilm, characterized by persistent inflammation that leads to the progressive destruction of the tooth-supporting apparatus. Periodontitis represents a major public health problem due to its high prevalence, contribution to tooth loss, and subsequent impairment of masticatory function, as well as its impact on aesthetics. Additionally, it is linked to social inequality, a reduced quality of life, and significant effects on overall health [1]. According to the World Health Organization (WHO), severe forms of periodontal disease affect approximately 19% of the global population, which corresponds to more than one billion cases in individuals over the age of 15 [2].

More than 50 diseases and conditions have been associated with PD, including cardiovascular diseases, Alzheimer’s disease, diabetes, rheumatoid arthritis, aspiration pneumonia, and cancer, including colorectal cancer (CRC) [3]. Table 1 presents epidemiological data on the association between PD and CRC.

CRC is the third most common type of cancer worldwide, with more than 1.9 million new cases diagnosed in 2022. It represents the second leading cause of cancer-related mortality, accounting for over 900,000 deaths annually, despite the availability of effective screening methods that could significantly reduce mortality from this disease [11].

Genetic susceptibility and family history [12] account for approximately 10% of human colorectal cancer cases, and the contribution of genetic factors [13] has also been demonstrated in periodontitis. In a study by Di Spirito et al., the existence of a genetic linkage between periodontitis and human colorectal cancer was assessed, identifying »leader« genes involved in the association between these two disorders. Table 2 provides an overview of the 12 “leader” genes and their genetic linkage between PD and CRC [14].

In terms of environmental factors, several play a crucial role, such as smoking, obesity, aging, and other risk factors. Studies show that chronic inflammation also plays a key role in the development of CRC [15]. PD is one of the most prevalent chronic inflammatory diseases in humans [16]. In 2010, PD ranked as the sixth most common disease condition globally [17]. The mechanism that connects periodontitis to colorectal cancer involves the dissemination of periodontal bacteria, their inflammatory products, and endotoxins to the intestine, creating a favorable environment for chronic inflammation and tumor promotion [18].

Two main pathways have been proposed for the spread of bacteria from the oral cavity to the intestine. The first is the hematogenous route, in which bacteria enter the bloodstream through oral lesions and subsequently reach the intestinal mucosa via circulation. The second is the enteral route, whereby bacteria survive passage through the stomach and colonize the intestine. Although the human body is equipped with defense mechanisms along this pathway—such as gastric acid neutralization and the colonization barrier against foreign microorganisms—these defenses can at times be compromised [19].

## 2. Red Complex Periodontal Pathogens and Their Role in Colorectal Carcinoma

In PD, dysbiosis occurs due to a shift in the subgingival microbiome, where Gram-negative bacteria begin to dominate over Gram-positive bacteria. The development of periodontal dysbiosis occurs over an extended period, gradually transforming the symbiotic relationship between the host and the microbiome into a pathogenic state [20].

Socransky and Haffajee found that in cases of periodontal disease, certain bacterial species are detected more frequently and in higher abundance compared to individuals with healthy periodontal tissues [21].

**Figure 1:**
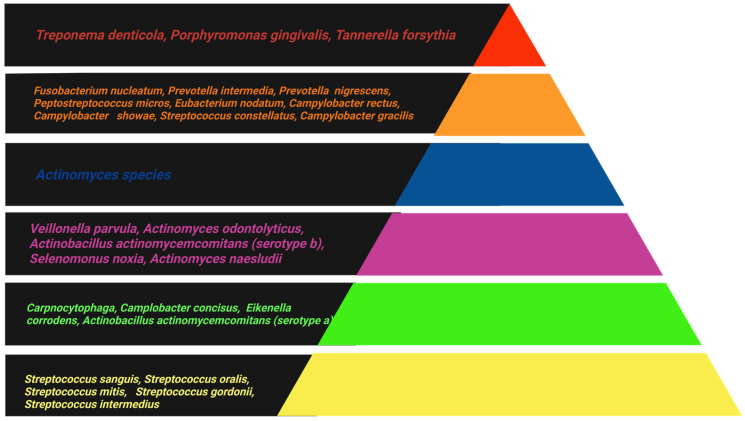
The association among subgingival species. The different colors in the triangle represent different bacterial complexes, which are frequently detected in association with one another [21]. (Created by BioRender).

In the 1998 study by Socransky and Haffajee, 13,000 plaque samples from 185 individuals were analyzed using cluster analysis and community ordination techniques to investigate associations between species within the biofilm. The study led to a shift in the understanding of periodontal infection, showing that it is bacterial complexes, rather than individual species, that are associated with either periodontal health or PD. This finding gave rise to the concept that co-dependence and synergy among different bacterial species contribute to the formation of specific complexes. Figure 1 represents the periodontal bacterial complexes described by this study [21].

Bacteria of the yellow complex, predominantly streptococci, and the green complex, including species of *Capnocytophaga*, are considered early colonizers in dental plaque and are associated with periodontal health. The orange complex consists of bacteria that colonize later, such as *Fusobacterium*, *Prevotella*, and *Campylobacter* species, which facilitate the colonization of the red complex either through binding sites or by creating an environment conducive to the growth of these pathogenic species. PD is most commonly associated with bacteria of the red complex, namely *Porphyromonas gingivalis*, *Tannerella forsythia*, and *Treponema denticola* [22,23].

## 3. Methods

This narrative review provides an overview of current knowledge on the potential role of red complex periodontal pathogens in the development of CRC. It highlights both clinical and experimental evidence linking *Porphyromonas gingivalis*, *Tannerella forsythia*, and *Treponema denticola* to CRC. A comprehensive literature search was performed in PubMed, Google Scholar, and ScienceDirect using the keywords: “periodontal disease”, “red complex”, “*P. gingivalis*”, “*T. forsythia*”, “*T. denticola*”, and “colorectal cancer”. The search covered studies published up to September 2025 with language restrictions to English and Slovenian. Titles and abstracts were screened for relevance, and full-text articles were evaluated according to predefined criteria. Inclusion criteria were original clinical, in vivo, or in vitro studies directly addressing the relationship between red complex periodontal pathogens and colorectal cancer. Reviews, case reports, and studies not focusing on the red complex were excluded. The final selection was based on methodological quality and relevance to the research question. The final search result included 12 studies that met the criteria.

## 4. Results of the Available Literature

### 4.1. Porphyromonas gingivalis

*Porphyromonas gingivalis* is a Gram-negative, non-spore-forming, non-motile, black-pigmented, asaccharolytic coccobacillus that is strongly associated with periodontal disease. In healthy periodontal tissues, it is detected only rarely and at very low levels. As a secondary colonizer, *Porphyromonas gingivalis* does not establish on clean tooth surfaces; instead, it adheres to host tissues and pre-existing microbial communities [24].

*Porphyromonas gingivalis* possesses a wide range of virulent factors. Among the most important are the lipopolysaccharides (LPS) of its Gram-negative cell wall, which are recognized by toll-like receptor 4 (TLR4) and activate both MyD88-dependent and -independent pathways, ultimately leading to nuclear factor-kappa B (NF-κB) activation and the production of pro-inflammatory cytokines. This process leads to the establishment of a pro-inflammatory microenvironment that promotes cancer development and progression [24,25]. Additional virulence factors include the capsule, fimbriae, hemagglutinins, hemolysins, collagenases, nucleases, fibrinolysins, membrane-associated proteases, fibroblast-activating factors, as well as cytotoxic factors targeting the mucosal epithelium, such as the cysteine protease gingipain [24].

In a study by Mu et al., the potential role of the virulence factor gingipain, a *Porphyromonas gingivalis*-associated factor, in the progression of CRC was examined. The authors demonstrated that gingipain is a key virulence determinant that facilitates the entry of *Porphyromonas gingivalis* into CRC cells and promotes their proliferation through the activation of the mitogen-activated protein kinase/extracellular signal-regulated kinase 1/2 (MAPK/ERK) signaling pathway [26].

Western blot analysis revealed that the total membrane (TM) and peptidoglycan (PDG) of *Porphyromonas gingivalis*, as well as viable bacteria, significantly upregulated Programmed cell death ligand 1 (PD-L1) expression in colon cancer cells. PD-L1 plays a crucial role in immune regulation by promoting the development of regulatory T cells and facilitating tumor immune evasion through the engagement of PD-1. Its upregulation is also linked to cancer progression, drug resistance, and metastasis. Inhibitors of nucleotide-binding oligomerization domain (NOD) proteins 1 and 1/2 (NOD1 and NOD1/2) suppressed PD-L1 induction, indicating that bacterial *Porphyromonas gingivalis* activates these receptors. Further, inhibition of Receptor-Interacting Protein Kinase 2 (RIP2) and MAPK pathways reduced PD-L1 expression, confirming their role in this process. These findings suggest that *Porphyromonas gingivalis* peptidoglycan drives PD-L1 upregulation via NOD1/2–RIP2–MAPK signaling, contributing to immune evasion in CRC [27].

Exogenous substances, such as pathogenic bacteria, can activate the innate (non-adaptive) immune response of the host, which may subsequently trigger a strong inflammatory reaction. Nod-like receptors, one of the three major classes of innate immune receptors, participate in the assembly of a large multiprotein complex known as the inflammasome, which responds to pathogens such as *Porphyromonas gingivalis* [28]. In their study, Wang et al. investigated how activation of the innate immune response—whose primary role is to recognize pathogen-associated molecular patterns (PAMPs) and foreign particles—may contribute to the development of CRC through stimulation by *Porphyromonas gingivalis*. It was demonstrated that *Porphyromonas gingivalis* reshapes the tumor–immune microenvironment by selectively expanding myeloid-derived immune cells and promoting an inflammatory milieu. Moreover, activation of the hematopoietic NOD-, LRR-, and pyrin domain-containing protein 3 (NLRP3) inflammasome has been demonstrated to be crucial for PG–induced colorectal cancer development [28].

Infection of CRC cells also influences the host immune response against tumor cells. *Porphyromonas gingivalis* induces a protumor phenotype by impairing iNKT (invariant natural killer T) cell cytotoxicity through upregulation of chitinase-3-like-protein-1 (CHI3L1) and by promoting neutrophil recruitment within the tumor microenvironment. iNKT cells, specialized T lymphocytes that recognize glycolipid antigens via CD1d, normally contribute to cancer immune surveillance and can kill CRC cells through the perforin–granzyme pathway. However, in CRC, the tumor microenvironment suppresses their antitumor functions, favoring immunosuppressive myeloid responses and metastasis. The study by Diaz-Basabe et al. suggests that *Porphyromonas gingivalis* further exacerbates this suppression, providing new insight into microbial contributions to immune evasion in CRC [29].

In a study by Yang Lu et al., the proteomic characteristics of CRC cells were investigated following infection with *Porphyromonas gingivalis*. The authors identified alterations in the expression of 335 proteins upon infection. Among these, the most notable change was the upregulation of ubiquitin carboxyl-terminal hydrolase L3 (UCHL3), a deubiquitinase with a well-established role in CRC progression. *Porphyromonas gingivalis*-induced UCHL3 may promote cancer progression by activating the NF-κB signaling pathway, which is mediated by the substrate protein GNG12, a guanine nucleotide-binding protein involved in various cellular signal transduction pathways. It has been demonstrated that GNG12 influences tumor immune evasion, cell proliferation, and migration, indicating that it is a potential oncoprotein in the tumorigenesis of colon cancer [30].

By using an AOM/DSS mouse model, Motosugi et al. demonstrated that oral administration of *Porphyromonas gingivalis* aggravates CRC severity. Microbiota profiling revealed that *Porphyromonas gingivalis* was significantly enriched in the mucosa-associated microbiota (MAM) but not in the luminal-associated microbiota (LAM) in both mouse and human samples. In vitro analyses revealed that *Porphyromonas gingivalis* exhibited a higher adhesion capacity to intestinal epithelial cells compared to *Prvotella intermedia*, mediated by strain-specific fimbriae and gingipains that facilitate disruption of the mucosal barrier and epithelial colonization. These findings suggest that *Porphyromonas gingivalis* contributes to CRC pathogenesis by facilitating MAM translocation, promoting strong epithelial adherence, and modulating the immune response [31].

Repeated oral exposure to *Porphyromonas gingivalis* altered the intestinal microbiota and induced immune-inflammatory responses in the gut, spleen, and liver. In *Porphyromonas gingivalis*-infected mice, microbial diversity was reduced, accompanied by an increased *Bacteroidetes-to-Firmicutes* ratio, a pattern linked to intestinal inflammation and liver disease. Inflammatory infiltration was observed in the gut, with elevated mRNA levels of TNF-α, IFN-γ, and IRF-1, indicating a predominant Th1 response. These findings suggest that oral *Porphyromonas gingivalis* disrupts gut microbial balance and promotes systemic inflammation, contributing to intestinal and hepatic pathology [32].

A positive infection of CRC cells with *Porphyromonas gingivalis* also affects overall patient survival. Wang et al. confirmed in two cohort studies that *Porphyromonas gingivalis* infection is associated with a poorer prognosis (survival) in CRC patients compared to those who are negative [28]. Similarly, Kerdreux et al. reported a positive association between fecal samples and worse cancer prognosis in *Porphyromonas gingivalis*-positive patients. However, unlike Wang et al., they did not observe a statistically significant difference in survival between patients with and without PG in the CRC tissue itself [33].

### 4.2. Treponema denticola

It belongs to the small or medium-sized oral spirochetes, which are isolated in increased quantities from the subgingival microbiota in PD. It can adhere to hydroxyapatite, epithelial cells, fibroblasts, as well as glycoproteins, fibronectin, and laminin. It exerts a cytotoxic effect on the epithelium and secretes proteolytic enzymes that degrade fibronectin, laminin, and collagen. Trypsin-like proteases act directly in a destructive manner on periodontal tissue and indirectly through tissue proteases, as well as by stimulating inflammatory mediators. It also secretes phospholipase, causing the hemolysis of erythrocytes. Furthermore, it inhibits the proliferation of fibroblasts and endothelial cells and prevents the activation of lymphocytes and the degranulation of neutrophils. In its outer membrane, some glycopeptides are likely involved in the adhesion of *treponemes* to human epithelium [24].

Nieminen et al. did a study where they examined the presence of Dentilisin: *Treponema denticola* chymotrypsin-like proteinase (Td-CTLP) in major oral and GI (orodigestive) tumors in vivo using immunohistochemistry and the ability to modulate immunomodulatory proteins: matrix metalloproteinases (MMPs), tissue inhibitor of MMPs (TIMPs), protease protein alpha-1-antichymotrypsin (α-1-AC), and complement C1q. Td-CTLP was detected in the adenocarcinoma of the colon, but not in every sample. They were mainly detected in granular deposits within the cytoplasm of epithelial cells in tumors. In vitro, Td-CTLP activated proMMP-8 and proMMP-9, fragmented TIMP-1 and TIMP-2, α-1-AC, and C1q, and enhanced the MMP–8–mediated degradation of type I and II collagens [34].

The study of Haheim et al., a prospective cohort study, investigated the relationship between antibody levels to specific oral bacteria and the risk of cancer over 17½ years. Low antibody levels to *Treponema denticola* were associated with increased risk of both colon cancer and bladder cancer. The study suggests that a low immune response to pathogenic oral bacteria may permit bacterial spread and contribute to the development of cancer. Factors such as chronic periodontal infection, genetic predisposition, and impaired IgG production may underline these associations [35].

### 4.3. Tannerella forsythia

*Tannerella forsythia* is an obligatory anaerobic, fusiform-shaped Gram-negative bacillus. It is non-pigmented and non-saccharolytic. The bacterium hydrolyzes esculin and gelatin, but it cannot ferment carbohydrates. Its metabolism depends on external sources of *N*-acetylmuramic acid, a component of bacterial cell walls, which it acquires through interactions with other bacteria capable of synthesizing this compound. *Tannerella forsythia* predominantly resides in dental plaque, where peptides and proteins derived from saliva, serum, and the surrounding microbial community are abundant. For growth, it requires peptides and possesses peptidase activity to utilize them [24].

Its pathogenicity relies heavily on proteolytic enzymes. The cysteine protease PrtH degrades large protein substrates, disrupts host cell adhesion, induces IL-8 production, and exhibits cytopathic effects by arresting cells in the G2 phase of the cell cycle. Additional proteases such as karylysins and mirolase degrade host defense molecules (e.g., LL-37, fibrinogen, hemoglobin), modulate complement activation, and promote neutrophil recruitment. These irreversible proteolytic processes contribute to tissue destruction, immune evasion, and bacterial persistence [36,37].

Other virulence factors include miropin, a serpin that inhibits neutrophil proteases; S-layer glycoproteins and BspA proteins, which facilitate adhesion to host tissues; surface lipoproteins that trigger the apoptosis of gingival fibroblasts; and glycosidases that hydrolyze host oligosaccharides, thereby supporting nutrient acquisition. Notably, accumulation of the toxic metabolite methylglyoxal during glucose metabolism may exacerbate tissue injury [36,37].

*Tannerella forsythia* also interacts synergistically with *Porphyromonas gingivalis*: while *Tannerella forsythia* produces succinate to support *Porphyromonas gingivalis* growth, *Porphyromonas gingivalis* provides peptides and amino acids through its proteolytic activity. Together, these pathogens contribute to the progression of periodontal disease [37].

Beyond periodontitis, epidemiological evidence suggests a role for *Tannerella forsythia* in gastrointestinal malignancies, particularly esophageal adenocarcinoma (EAC). Proposed mechanisms include chronic inflammation induced by the induction of IL-1β, IL-6, and TNF-α; activation of the TLR/NF-κB signaling pathways; and dysbiosis of the oral microbiome. In combination with *Porphyromonas gingivalis*, *Tannerella forsythia* also promotes the overexpression of GLUT1/GLUT4 through TNF-α and ROS signaling, a hallmark of tumor aggressiveness [37].

While direct links between *Tannerella forsythia* and CRC remain unconfirmed, mechanistic similarities to esophageal carcinogenesis suggest its potential contribution to gastrointestinal tumorigenesis warrants further investigation. Table 3 provides an overview of studies examining the role of red complex bacteria in colorectal cancer.

## 5. Conclusions, Future Directions, and Clinical Implications

The human microbiome plays a crucial role in the development of the body and contributes to numerous beneficial processes that benefit the host (the human). The relationship is mutually beneficial (symbiotic). The state in which bacteria are in homeostatic balance is called EUBIOSIS [38]. Occasionally, the balance of microorganisms is disrupted, and the synergistic activity breaks down, which can lead to the development of disease. This process is called dysbiosis [39]. Dysbiosis is a change in the bacterial composition of the biofilm, characterized by the overgrowth of pathogens, which leads to an undesirable immune response of the host [1].

Oral dysbiosis, or altered oral microbiota, was recently linked to a significantly positive association with GI cancers (esophageal, pancreatic, and CRC). Dysbiosis of the oral microbiota is caused by two major oral pathologies: dental caries and periodontitis, also known as PD. Increased colonization of subgingival Gram-negative anaerobic bacteria results in deep pockets, loss of clinical attachment, and bleeding on probing (BOP), all corresponding to PD. It is widely accepted that *Treponema denticola*, *Porphyromonas gingivalis*, and *Tannerella forsythia* form a bacterial consortium, often referred to as the “red complex”, that is strongly associated with the clinical progression of chronic periodontitis. The unifying features of the red complex bacteria are their extracellular proteolytic activity, their complex anaerobic fermentation of amino acids, the production of toxic metabolites, and the presence of outer membrane vesicles, which are known to contribute to the carcinogenesis of GI cancers [3,20,40].

Although the current body of evidence highlights potential links between red complex periodontal pathogens and CRC, many unanswered questions remain. Most available studies have concentrated on *Porphyromonas gingivalis*, while the roles of *Treponema denticola* and *Tannerella forsythia* are far less understood. Future research should therefore prioritize mechanistic investigations into the diverse virulent factors of these bacteria and their influence on CRC. Longitudinal cohort studies combining microbial profiling, host immune responses, and genetic susceptibility markers would provide more definitive evidence for causality.

The potential clinical utility of periodontal pathogens as biomarkers for CRC also warrants further exploration. Oral and gut microbial signatures, particularly when combined with salivary, fecal, and blood-based analyses, may support the development of non-invasive screening tools for CRC risk prediction and prognosis. At the same time, microbial virulence factors such as gingipains or Td-CTLP present attractive therapeutic targets that could inform precision medicine strategies. Periodontal treatment, probiotic supplementation, and microbiome-modulating therapies may emerge as preventive or adjunctive interventions aimed at reducing systemic inflammation and CRC risk.

From a clinical perspective, routine periodontal care could be integrated into oncology prevention programs, especially for individuals with established risk factors for CRC. Interdisciplinary collaboration is essential to achieve this goal. Periodontology, oncology, and microbiome research should converge through multicenter studies, shared biobanks, and advanced bioinformatic analyses to identify microbial signatures predictive of cancer development. Such collaborations could foster the creation of microbiome-informed risk models and personalized preventive approaches.

Looking forward, a clearer understanding of the oral–gut microbiome axis has the potential to reshape both periodontal and oncological practice. Integration of oral health into systemic disease prevention strategies may become a cornerstone of comprehensive patient care. Addressing periodontal pathogens may not only preserve oral health but also contribute meaningfully to reducing the global burden of CRC.

## Figures and Tables

**Table 1:** Overview of epidemiological studies investigating the link between periodontal disease (PD) and colorectal cancer (CRC).

Study	Population	Periodontal Status	Key Findings
Population-based case–control study [4]	Residents of Montreal Island or Laval, aged 40–80; 348 CRC patients and 310 controls	self-reported (questionnaire)	The CRC in persons with a positive history of PD was 1.45 times higher than in those with a negative history of PD.
Cross-sectional, retrospective study [5]	Patients who received colonoscopy as a part of routine health check-ups; 2504 patients enrolled; CHA Bundang Medical Center, Korea	Dental examination	Prevalence of proximal neoplasma was higher in the PD group (25%) vs. the control group (12.3%). Parodontitis was not an overall risk factor; however, it was associated with an increased risk for proximal colon neoplasms.
NHS-prospective cohort study [6]	77,443 female nurses aged 30–55 (18 years follow-up), from 11 states in the US	Self-reported (history of periodontal disease and number of natural teeth)	Women with fewer teeth, possibly moderate or severe periodontal disease, might be at a modestly increased risk of developing CRC.
3 cohort studies; case/control [7]	825 case/control (SMHS/SWHS) and 238/2258 (SCCS) *	Questioner (number of teeth)	Tooth loss was not associated with increased risk of CRC.
Prospective cohort study [8]	7466 participants in the Atherosclerosis Risk in Communities study cohort; age 44–66 years from Jackson, Mississippi, Washington County, Maryland; Minneapolis, Minnesota; and Forsyth County, North Carolina	Periodontal examination	An increased risk of total cancer was observed for severe periodontitis.
Nationwide retrospective cohort study [9]	A total of approximately 106,487 individuals with newly diagnosed PD and 106,487 age-matched and sex-matched patients without PD from 2000 to 2002 were identified from Taiwan’s National Health Insurance Research Database (NHIRD).	They relied on administrative/insurance data (diagnosis codes + visit frequency) to infer the presence and severity of periodontal disease.	The incidence of CRC was significantly higher in patients with PD than in those without PD.
A population-based, retrospective cohort study [10]	The Korean National Health Insurance Cohort Database was obtained between January 2003 and December 2015; it included 713,201 individuals without a history of cancer who were followed up to 10 years	They relied on administrative/insurance data (diagnosis codes + visit frequency) to infer the presence and severity of periodontal disease.	The cumulative incidence of cancer in the periodontitis group was 2.2 times higher than that in the control group. The periodontitis group had an increased risk of total cancer compared to the control group.

* SCCS—Southern Community Cohort Study, SMHS—Shanghai Men’s Health Study, SWHS—Shanghai Women’s Health Study.

**Table 2:** Summary of the 12 leader genes identified in the genetic linkage between periodontal disease (PD) and colorectal carcinoma (CRC), based on the study by Di Spirito et al. [14].

Gene	Main Function/Biological Process	Putative Pathogenic Mechanism in the Linkage
*CTNNB1*	Cell signaling (Wnt pathway), cell growth, and adhesion	Cell cycle dysregulation: abnormal proliferation, transformation in the colon; disturbed tissue homeostasis in the periodontium.
*FOS*	Transcription factor; forms AP-1 with *JUN*; regulates proliferation and differentiation	Cell cycle regulation promotes proliferation and may also contribute to inflammation via AP-1-mediated transcription.
*JUN*	Part of the AP-1 transcription factor (with *FOS*); regulates genes in proliferation, apoptosis, and differentiation.	Cell cycle dysregulation promotes proliferative and inflammatory signaling.
*GRB2*	Adaptor protein in signaling (e.g., binding EGFR, etc.)	Cell signaling dysregulation leading to abnormal proliferation and possibly impaired tissue repair.
*PIK3CA*	Catalytic subunit of *PI3K*; cell proliferation and survival	Cell cycle dysregulation, which can enhance the survival of cells, may possibly contribute to malignancy in the colon.
*PIK3R1*	Regulatory subunit of PI3K; controls PI3K activity/signaling	Cell signaling dysregulation influences proliferation, possibly inflammation.
*IL6*	Cytokine: an immunoinflammatory mediator	Immuno-inflammatory response: sustained inflammation, systemic effects that may favor the tumor environment.
*IL1B*	Pro-inflammatory cytokine	Immuno-inflammatory response: persistent inflammation, possibly promoting DNA damage, cell proliferation, and tumorigenesis.
*IL4*	Cytokine modulates immune response (Th2)	Immuno-inflammatory modulation: imbalance may favor tumor-promoting inflammation or immune suppression.
*IL10*	Anti-inflammatory cytokine; regulates immune suppression	Immuno-inflammatory response: when anti-inflammatory regulation fails, inflammation becomes chronic, aiding carcinogenesis.
*RELA*	Transcription factor; major part of NF-κB complex; regulates pro-inflammatory genes.	Immuno-inflammatory response: drives expression of cytokines, etc.; links inflammation and possibly proliferation signals.
*CBL*	E3 ubiquitin ligase; modulates proteasomal degradation of proteins; impacts signaling and NF-κB	Immuno-inflammatory response; possibly via dysregulation of protein turnover, NF-κB activation.

**Table 3:** Summary of studies investigating the impact of red complex bacteria on colorectal cancer (CRC).

Red Complex Bacteria	Virulence Factor	Mechanism of Action	Local Effect in CRC	Known Systemic Effect
*Porphyromonas gingivalis*	Peptidoglycan [27]	Upregulation of PD-L1 via NOD1/2–RIP2–MAPK signaling [27], impairing iNKT cell cytotoxicity through upregulation of CHI3L1 [29].	**Tumor immune evasion**	Cancer progression, drug resistance, and metastasis
*Porphyromonas gingivalis*	Strain-specific fimbriae and gingipains [31]	Reduced microbial diversity; an increased *Bacteroidetes-to-Firmicutes* ratio [32]., The gut microbiome changed, favoring *Erysipelotrichaceae* [28] disruption of the mucosal barrier and epithelial colonization [31].	**Changes in the gut microbiota**	Progression of CRC
*Porphyromonas gingivalis*	NLRP3 expression within the hematopoietic compartment [28]	Increased inflammatory cells; mRNA levels of TNF-α, IRF-1, and IFN-γ significantly increased [32]. Increase accumulation of the infiltrating myeloid cell line [28]. Increase levels of TNFα, IL6 in IL1β [28].	**Intestinal inflammation/pro-inflammatory microenvironment**	Promotes systemic inflammation
*Porphyromonas gingivalis*	gingipain [26]	Activation of MAPK/ERK signaling pathways [26]. Upregulation of UCHL3; GNG12-activated NF-κB signal pathway [30].	**Promoting CRC Cell proliferation**	Tumor progression, metastasis
*Treponema denticola*	Td-CTLP	Activated proMMP-8 and proMMP-9, fragmented TIMP-1 and TIMP-2, α-1-AC, and C1q, and enhanced the MMP–8–mediated degradation of type I and II collagens in vitro; detected as granular deposits within the cytoplasm of epithelial cells in tumors [34].	**Pro-inflammatory and tissue-destructive environment**	Tumor progression
*Tanarella forsythia* *		Induction of IL-1β, IL-6, and TNF-α; activation of the TLR/NF-κB signaling pathways [37].	**Chronic inflammation**	
*Tanarella forsythia* *	In combination with *Porphyromonas gingivalis*	Overexpression of GLUT1/GLUT4 through TNF-α and ROS signaling [37].	**Fueling the growth and survival of the tumor**	Tumor progression

* The study focuses on gastrointestinal malignancy, particularly oesophageal adenocarcinoma. There are no studies linking *Tannerella forsythia* and CRC. UCHL3—ubiquitin carboxyl-terminal hydrolase L3; GNG12—guanine nucleoti-de-binding protein; MAPK/ERK—mitogen-activated protein kinase/extracellular signal-regulated kinase 1/2 signaling pathway; TNF-α—tumor necrosis factor α; IL6—interleukin 6; IL1β—interleukin 1β; NLRP3 inflammasome: pyrin domain-containing protein 3 inflammasome; iNKT—invariant natural killer T; PD-L1: Programmed Death-Ligand 1 (CD274); CHI3L1—chitinase-3-like-protein-1; IRF-1: Interferon Regula-tory Factor 1; IFN-γ: interferon gamma; NOD—nucleotide-binding oligomerization domain protein; RIP2—Receptor-Interacting Protein Kinase 2.

## Data Availability

No new data were created or analyzed in this study. Data sharing is not applicable to this article.

## Abbreviations

The following abbreviations are used in this manuscript:

PD	Periodontal disease
WHO	World Health Organization
CRC	Colorectal Carcinoma
